# Prescription Opioid Misuse and Use of Alcohol and Other Substances Among High School Students — Youth Risk Behavior Survey, United States, 2019

**DOI:** 10.15585/mmwr.su6901a5

**Published:** 2020-08-21

**Authors:** Christopher M. Jones, Heather B. Clayton, Nicholas P. Deputy, Douglas R. Roehler, Jean Y. Ko, Marissa B. Esser, Kathryn A. Brookmeyer, Marci Feldman Hertz

**Affiliations:** ^1^Office of the Director, National Center for Injury Prevention and Control, CDC; ^2^Division of Adolescent and School Health, National Center for HIV/AIDS, Viral Hepatitis, STD, and TB Prevention, CDC; ^3^Epidemic Intelligence Service, CDC; ^4^Division of Overdose Prevention, National Center for Injury Prevention and Control, CDC; ^5^Division of Reproductive Health, National Center for Chronic Disease Prevention and Health Promotion, CDC; ^6^Division of Population Health, National Center for Chronic Disease Prevention and Health Promotion, CDC; ^7^Division of STD Prevention, National Center for HIV/AIDS, Viral Hepatitis, STD, and TB Prevention, CDC

## Abstract

Adolescence is an important period of risk for substance use initiation and substance use–related adverse outcomes. To examine youth substance use trends and patterns, CDC analyzed data from the 2009–2019 Youth Risk Behavior Survey. This report presents estimated prevalence of current (i.e., previous 30-days) marijuana use, prescription opioid misuse, alcohol use, and binge drinking and lifetime prevalence of marijuana, synthetic marijuana, cocaine, methamphetamine, heroin, injection drug use, and prescription opioid misuse among U.S. high school students. Logistic regression and Joinpoint analyses were used to assess 2009–2019 trends. Prevalence of current and lifetime substance use by demographics, frequency of use, and prevalence of co-occurrence of selected substances among students reporting current prescription opioid misuse are estimated using 2019 data. Multivariable logistic regression analysis was used to determine demographic and substance use correlates of current prescription opioid misuse. Current alcohol, lifetime cocaine, methamphetamine, heroin, and injection drug use decreased during 2009–2019. Lifetime use of synthetic marijuana (also called synthetic cannabinoids) decreased during 2015–2019. Lifetime marijuana use increased during 2009–2013 and then decreased during 2013–2019. In 2019, 29.2% reported current alcohol use, 21.7% current marijuana use, 13.7% current binge drinking, and 7.2% current prescription opioid misuse. Substance use varied by sex, race/ethnicity, grade, and sexual minority status (lesbian, gay, or bisexual). Use of other substances, particularly current use of alcohol (59.4%) and marijuana (43.5%), was common among students currently misusing prescription opioids. Findings highlight opportunities for expanding evidence-based prevention policies, programs, and practices that aim to reduce risk factors and strengthen protective factors related to youth substance use, in conjunction with ongoing initiatives for combating the opioid crisis.

## Introduction

Substance use and associated adverse outcomes contribute to substantial morbidity, mortality, and economic costs to society each year in the United States (*1*). Data from national surveys indicate the majority of adolescents will engage in some form of substance use before they graduate from high school (https://www.samhsa.gov/data/report/2018-nsduh-detailed-tables). During adolescence, areas of the brain associated with emotional responses and reward systems develop before those associated with executive functioning, judgement, and decision making (*2*). This uneven maturation results in increased susceptibility for engaging in risky and impulsive behaviors, including substance use, and increases vulnerability to reinforcing and rewarding effects of substances (*2*,*3*). Preventing or delaying substance use initiation among youths can reduce later risk for substance use and substance use disorders (*1*,*3*,*4*). Beyond the individual negative effects of substance use during youth and into adulthood, substance use among youths also increases the likelihood for negative consequences that affect peers, families, and communities (*5*). Youth substance use is associated with increased risk for delinquency, academic underachievement, teenage pregnancy, sexually transmitted diseases, perpetrating or experiencing violence, injuries, and mental health problems (*1*,*3*–*6*).

As the United States confronts its decades-long opioid overdose epidemic (*1*,*2*), preventing opioid misuse among youth is a public health imperative. Previous research has documented that misuse of prescription opioids among youths is associated with multiple adverse health outcomes and risk behaviors, including use of alcohol and other illicit drugs, injection drug use, suicidal ideation, youth violence, delinquency, having four or more lifetime sexual partners, not using a condom at last sexual intercourse, increased risk for acquisition of human immunodeficiency virus infection and sexually transmitted diseases (*6*), and increasing overdoses (*7*). Studies also have demonstrated that prescription opioid misuse among youths is strongly linked with subsequent initiation and use of heroin and increased risk for injecting prescription opioids and developing an opioid use disorder (*8*–*10*).

Preventing substance use among youths is necessary because of the health and social effects of youth substance use. To inform substance use prevention initiatives and to improve understanding of youth substance use patterns, including misuse of prescription opioids and other substances, this analysis 1) examines trends and patterns in substance use among high school students overall and by demographic characteristics, 2) characterizes the frequency of use of specific substances among high school students, 3) explores co-occurring substance use among high school students who misuse prescription opioids, and 4) examines the demographic and substance use correlates of prescription opioid misuse among high school students. Findings from this analysis can help inform efforts by public health practitioners, clinicians, and the substance use prevention community to expand the implementation of evidence-based prevention policies, programs, and practices that aim to reduce risk factors and strengthen protective factors related to youth substance use.

## Methods

### Data Source

This report includes data from CDC’s 2009–2019 Youth Risk Behavior Survey (YRBS), a cross-sectional, school-based survey conducted biennially since 1991. Each survey year, CDC collects data from a nationally representative sample of public- and private-school students in grades 9–12 in the 50 U.S. states and the District of Columbia. Additional information about YRBS sampling, data collection, response rates, and processing is available in the overview report of this supplement (*11*). The prevalence estimates for all substance use questions for the overall study population and by sex, race/ethnicity, grade, and sexual orientation are available at https://nccd.cdc.gov/youthonline/App/Default.aspx. The full YRBS questionnaire is available at https://www.cdc.gov/healthyyouth/data/yrbs/pdf/2019/2019_YRBS-National-HS-Questionnaire.pdf.

### Measures

This report addresses four current (i.e., previous 30 days before the survey) and seven lifetime substance use behaviors. The four current substance use behaviors include 1) marijuana use (ascertained by the question, “During the past 30 days, how many times did you use marijuana?”), 2) alcohol use (“During the past 30 days, on how many days did you have at least one drink of alcohol?”), 3) binge drinking (“During the past 30 days, on how many days did you have 4 or more drinks of alcohol in a row, that is, within a couple of hours [if you are a female] or 5 or more drinks of alcohol in a row, that is, within a couple of hours [if you are a male]?”), and 4) prescription opioid misuse (“During the past 30 days, how many times have you taken prescription pain medicine without a doctor’s prescription or differently than how a doctor told you to use it?”). The current prescription opioid misuse question is new for the 2019 YRBS, providing opportunities to explore substance use patterns and individual characteristics associated with this variable for the first time.

The seven lifetime substance use behaviors include 1) marijuana use (“During your life, how many times have you used marijuana?”), 2) synthetic marijuana (also called synthetic cannabinoids) use (“During your life, how many times have you used synthetic marijuana?”), 3) cocaine use (“During your life, how many times have you used any form of cocaine, including powder, crack, or freebase?”), 4) methamphetamine use (“During your life, how many times have you used methamphetamines [also called speed, crystal meth, crank, ice, or meth]?”), 5) heroin use (“During your life, how many times have you used heroin [also called smack, junk, or China White]?”), 6) prescription opioid misuse (“During your life, how many times have you taken prescription pain medicine without a doctor's prescription or differently than how a doctor told you to use it?”), and 7) injection drug use (“During your life, how many times have you used a needle to inject any illegal drug into your body?”). 

Substance use behaviors were dichotomized to indicate current or lifetime use versus no use. With three exceptions, frequency of use for each substance was categorized as 1–2 times, 3–9 times, 10–39 times, or ≥40 times. Frequency of current alcohol use and current binge drinking were categorized as 1–2 days, 3–9 days, 10–19 days, or ≥20 days. For injection drug use, frequency of use was categorized as 1 time or ≥2 times.

Four demographic characteristics were included in the analysis: sex (male or female), race/ethnicity (non-Hispanic white [white], non-Hispanic black [black], Hispanic, or other), grade (9/10 or 11/12), and sexual identity (heterosexual; lesbian, gay, or bisexual; or not sure). Students reporting “other” race/ethnicity are included in all analyses; however, data are not presented for that group because of limited interpretability.

### Analysis

First, annual prevalence of each substance use behavior was estimated for all years with available data. Second, to identify temporal trends, logistic regression analyses were used to model linear and quadratic time effects while controlling for sex, grade, and racial/ethnic group changes over time; for significant quadratic time effects, Joinpoint software was used to identify the year the trend changed direction (*11*). Trends were assessed during 2009–2019 for current alcohol, current marijuana, lifetime marijuana, lifetime cocaine, lifetime methamphetamine, lifetime heroin, and lifetime injection drug use. Synthetic marijuana use was first assessed by YRBS in 2015; therefore, trend analysis for this variable was conducted for 2015–2019. Third, to identify 2-year changes in substance use behaviors, prevalence estimates from 2017 and 2019 were compared by using *t*-tests; changes were considered statistically different if the p value was <0.05.

Four additional analyses were conducted by using 2019 YRBS data only. First, prevalence estimates and associated 95% confidence intervals (CIs) for each substance use behavior were calculated by demographic characteristics. Statistically significant pairwise differences between demographic groups for each of the substance use behaviors were determined by *t*-tests; differences were considered statistically significant if the p value was <0.05. Second, to examine frequency of use among students who reported engaging in each substance use behavior, prevalence estimates and 95% CIs of students reporting each frequency of use category were calculated. Third, prevalence estimates of co-occurring use of selected substances among students reporting current prescription opioid misuse were estimated. Finally, multivariable logistic regression analysis was used to determine demographic and substance use correlates of current prescription opioid misuse. Because the use of one substance is generally strongly associated with use of one or more other substances, it is important to account for multiple substance use behaviors during the modeling process. Therefore, all demographic and substance use variables were included in a single model to examine the independent effect of each variable on current prescription opioid misuse. This modeling strategy is consistent with previous research examining substance use behaviors among youths (*6*).

To improve model stability during multivariable analyses, three composite substance use variables were created. A composite variable regarding alcohol consumption was created with three levels: 1) no previous 30-day use, 2) previous 30-day use (current drinking but no binge drinking), and 3) previous 30-day binge alcohol use. A marijuana composite variable also was created with three levels: 1) no lifetime use, 2) lifetime use but no previous 30-day use, and 3) previous 30-day use. A composite lifetime use of cocaine, heroin, or methamphetamine variable was created by combining answers of “1 or more times” for each of the three constituent variables. Because substance use variables are known to be highly correlated with each other, the Variance Inflation Factor was used to assess multicollinearity. None was observed (i.e., no values >10).

Adjusted prevalence ratios (aPRs) and corresponding 95% CIs were calculated; estimates were considered statistically significant if the 95% CI did not include 1.0. All analyses were conducted by using SAS-callable SUDAAN (version 11.0.1; RTI International) to account for survey weights and the complex sample design of the YRBS. No imputation methods were used for data that were missing.

## Results

Substance use was common among U.S. high school students during 2019 and varied by substance, year, and demographic groups (Table 1). Among current substance use measures, the highest prevalence estimates were for alcohol (29.2%) and marijuana use (21.7%). Current binge drinking was reported by 13.7% of high school students, and 7.2% reported current prescription opioid misuse. Among lifetime use measures, marijuana use was reported by 36.8% of high school students, followed by misuse of prescription opioids (14.3%) and use of synthetic marijuana (7.3%), cocaine (3.9%), methamphetamine (2.1%), or heroin (1.8%). Lifetime injection drug use was reported by 1.6% of high school students.

**TABLE 1:** Prevalence of and trends in prevalence of lifetime and current use of specific substances and use behaviors among high school students — Youth Risk Behavior Survey, United States, 2009–2019

Behavior	Prevalence						Linear change*	Quadratic change*	Change from 2017 to 2019^†^
	2009	2011	2013	2015	2017	2019			
**Current use** ^§^									
Marijuana	20.8	23.1	23.4	21.7	19.8	21.7	No change	No change	No change
Alcohol	41.8	38.7	34.9	32.8	29.8	29.2	Decreased 2009–2019	No change	No change
Binge drinking	—	—	—	—	13.5	13.7	NA^¶^	NA^¶^	No change
Prescription opioid misuse	—	—	—	—	—	7.2	NA^¶^	NA^¶^	NA^¶^
**Lifetime use**									
Marijuana	36.8	39.9	40.7	38.6	35.6	36.8	No change	Increased 2009–2013 Decreased 2013–2019	No change
Cocaine	6.4	6.8	5.5	5.2	4.8	3.9	Decreased 2009–2019	No change	No change
Methamphetamine	4.1	3.8	3.2	3.0	2.5	2.1	Decreased 2009–2019	No change	No change
Heroin	2.5	2.9	2.2	2.1	1.7	1.8	Decreased 2009–2019	No change	No change
Injection drug use	2.1	2.3	1.7	1.8	1.5	1.6	Decreased 2009–2019	No change	No change
Synthetic marijuana	—	—	—	9.2	6.9	7.3	Decreased 2015-2019	NA^¶^	No change
Prescription opioid misuse	—	—	—	—	14.0	14.3	NA^¶^	NA^¶^	No change

**Abbreviation**: NA = not available.

* Based on trend analyses by using a logistic regression model controlling for sex, race/ethnicity, and grade (p<0.05).

^†^ Based on *t*-test analysis (p<0.05).

^§^ Previous 30 days before the survey.

^¶^ Insufficient years of data to assess trends.

Trend data were available for eight of the 11 substance use measures included in the analyses. Among these measures, current alcohol use, lifetime cocaine, lifetime methamphetamine, lifetime heroin, and lifetime injection drug use decreased during 2009–2019. Lifetime use of synthetic marijuana decreased during 2015–2019. The prevalence of lifetime marijuana use increased during 2009–2013 (36.8%–40.7%) and then decreased during 2013–2019 (40.7%–36.8%). No statistically significant changes from 2017 to 2019 were observed for any of the substance use behaviors.

Compared with females, males had a significantly higher prevalence of lifetime use of cocaine (4.9% versus 2.7%), methamphetamine (2.7% versus 1.5%), heroin (2.3% versus 1.0%), and injection drug use (2.1% versus 1.1%) (Table 2). Compared with males, females had a significantly higher prevalence of current alcohol use (31.9% versus 26.4%), binge drinking (14.6% versus 12.7%), current prescription opioid misuse (8.3% versus 6.1%), and lifetime prescription opioid misuse (16.1% versus 12.4%). Among racial/ethnic groups, notable differences in prevalence estimates were identified for current use of alcohol, binge drinking, current prescription opioid misuse, and lifetime use of cocaine, methamphetamine, heroin, injection drug use, and synthetic marijuana. However, no clear pattern emerged. For example, the prevalence of current prescription opioid misuse was significantly lower among white students (5.5%) compared with black (8.7%) or Hispanic students (9.8%). Conversely, the prevalence of current alcohol use was lower among black students (16.8%) compared with white (34.2%) or Hispanic students (28.4%).

**TABLE 2:** Prevalence of lifetime and current use of specific substance and use behaviors among high school students, by demographic characteristics — Youth Risk Behavior Survey, United States, 2019

Behavior	Sex		Race/Ethnicity			Grade		Sexual identity		
	Male (n = 6,641) % (95% CI)	Female (n = 6,885) % (95% CI)	White, non-Hispanic (n = 6,668) % (95% CI)	Black, non-Hispanic (n = 2,040) % (95% CI)	Hispanic (n = 3,038) % (95% CI)	9/10 (n = 7,354) % (95% CI)	11/12 (n = 6,172) % (95% CI)	Heterosexual (n = 10,853) % (95% CI)	LGB (n = 1,531) % (95% CI)	Not sure (n = 591) % (95% CI)
Current use*										
Marijuana	22.5 (20.6–24.5)	20.8 (18.7–23.1)	22.1 (19.9–24.6)	21.7 (19.1–24.5)	22.4 (20.4–24.6)	17.1 (15.5–18.8)	26.6^†^ (23.6–29.7)	20.9 (19.0–23.0)	31.1^§^ (27.4–35.1)	19.5^¶^ (14.8–25.3)
Alcohol	26.4 (24.4–28.6)	31.9^**^ (29.6–34.3)	34.2 (31.7–36.8)	16.8^††^ (13.5–20.7)	28.4^††,§§^ (26.1–30.8)	22.8 (20.6–25.2)	36.0^†^ (33.8–38.3)	28.8 (26.8–30.8)	33.9^§^ (29.8–38.2)	25.3^¶^ (20.0–31.4)
Binge drinking	12.7 (11.0–14.6)	14.6^**^ (13.2–16.2)	17.3 (15.1–19.7)	6.2^††^ (4.2–9.2)	12.4^††,§§^ (11.0–14.0)	8.9 (7.4–10.7)	18.8^†^ (17.0–20.8)	13.4 (12.0–15.0)	15.6 (12.8–18.8)	13.1 (9.0–18.8)
Prescription opioid misuse	6.1 (5.3–7.1)	8.3^**^ (7.0–9.9)	5.5 (4.4–6.9)	8.7^††^ (6.5–11.6)	9.8^††^ (8.2–11.6)	7.0 (5.8–8.4)	7.3 (6.1–8.8)	6.4 (5.4–7.5)	12.0^§^ (9.6–14.9)	11.5^§^ (8.2–15.9)
**Lifetime use**										
Marijuana	37.0 (34.2–40.0)	36.5 (34.1–38.9)	36.8 (33.9–39.8)	37.5 (34.0–41.1)	39.2 (36.5–41.9)	29.2 (26.7–31.8)	44.8^†^ (41.5–48.2)	36.0 (33.3–38.7)	49.6^§^ (45.1–54.1)	27.5^§,¶^ (22.4–33.3)
Cocaine	4.9 (4.2–5.8)	2.7^**^ (2.0–3.7)	2.9 (2.2–3.7)	4.0 (2.7–5.9)	5.6^††^ (4.5–6.9)	2.8 (2.0–3.7)	5.0^†^ (4.1–6.1)	3.3 (2.7–4.0)	7.0^§^ (4.8–10.1)	7.6^§^ (4.3–12.9)
Methamphetamine	2.7 (2.1–3.4)	1.5^**^ (1.0–2.2)	1.2 (0.9–1.6)	3.8^††^ (2.4–6.0)	2.7^††^ (1.8–4.0)	1.5 (1.0–2.3)	2.6^†^ (1.9–3.3)	1.5 (1.2–1.9)	5.0^§^ (3.1–7.9)	6.1^§^ (3.4–10.8)
Heroin	2.3 (1.8–3.1)	1.0^**^ (0.6–1.8)	0.9 (0.6–1.2)	3.4^††^ (2.2–5.3)	2.4^††^ (1.5–3.9)	1.6 (1.0–2.5)	1.8 (1.3–2.5)	1.2 (0.9–1.6)	3.8^§^ (2.1–7.0)	6.2^§^ (3.4–11.0)
Injection drug use	2.1 (1.5–2.9)	1.1^**^ (0.6–1.9)	0.8 (0.6–1.2)	2.9^††^ (1.5–5.5)	2.5^††^ (1.8–3.5)	1.6 (1.1–2.3)	1.5 (1.0–2.4)	1.1 (0.8–1.6)	3.5^§^ (2.1–5.7)	5.1^§^ (2.5–10.2)
Synthetic marijuana	7.2 (6.2–8.4)	7.4 (6.2–8.7)	6.7 (5.6–8.0)	5.7 (4.4–7.4)	9.8^††,§§^ (8.6–11.3)	6.2 (5.3–7.3)	8.3^†^ (7.2–9.7)	6.7 (5.8–7.7)	11.6^§^ (9.0–14.7)	10.4 (6.9–15.5)
Prescription opioid misuse	12.4 (11.0–14.1)	16.1^**^ (14.1–18.4)	12.7 (10.9–14.7)	15.3 (12.9–18.1)	16.0 (13.5–18.8)	13.6 (11.9–15.5)	14.9 (13.2–16.7)	12.7 (11.2–14.4)	23.9^§^ (19.9–28.3)	19.1^§^ (14.6–24.5)

**Abbreviations:** CI = confidence interval; LGB = lesbian, gay, or bisexual.

* Previous 30 days before the survey.

^†^ Significantly different from 9/10 grade students, based on *t*-test analysis (p<0.05).

^§^ Significantly different from heterosexual students, based on *t*-test analysis (p<0.05).

^¶^Significantly different from lesbian, gay, or bisexual students, based on *t*-test analysis (p<0.05).

^**^ Significantly different from male students, based on *t*-test analysis (p<0.05).

^††^ Significantly different from white students, based on *t*-test analysis (p<0.05).

^§§^ Significantly different from black students, based on *t*-test analysis (p<0.05).

Approximately half of the substance use behaviors varied substantially by grade, with consistently higher prevalence among 11th- and 12th-grade students compared with 9th- and 10th-grade students for current marijuana use, current alcohol use and binge drinking, lifetime marijuana use, lifetime cocaine use, lifetime methamphetamine use, and lifetime synthetic marijuana. Prevalence of all but one of the substance use behaviors (i.e., binge drinking) varied considerably by sexual identity. Students who identified as lesbian, gay, or bisexual had a higher prevalence of all substance use behaviors, except binge drinking, compared with students who identified as heterosexual. Similarly, students who identified as not sure of their sexual identity also had higher prevalence of approximately half of the substance use behaviors compared with heterosexual students, including current prescription opioid misuse, lifetime cocaine use, lifetime methamphetamine use, lifetime heroin use, lifetime injection drug use, and lifetime prescription opioid misuse. However, students who identified as not sure of their sexual identity had lower prevalence of certain substance use behaviors compared with students identifying as lesbian, gay, or bisexual, including current marijuana use, current alcohol use, and lifetime marijuana use.

Frequency of use (i.e., number of times used or number of days used) varied across specific substance use behaviors (Table 3). Among students reporting marijuana use during the 30 days before the survey (i.e., current use), 18.0% reported using it ≥40 times; 23.5%, 10–39 times; 21.8%, 3–9 times; and 36.7%, 1–2 times. For current prescription opioid misuse, 9.8% reported misuse ≥40 times; 13.7%, 10–39 times; 23.3%, 3–9 times, and 53.2%, 1–2 times. Among students reporting lifetime use of specific substances, marijuana had the highest percentage of students reporting use ≥40 times (33.6%), followed by heroin (32.9%), methamphetamine (27.9%), and cocaine (16.1%). Lifetime prescription opioid misuse and lifetime synthetic cannabinoid use were the two substances with the highest percentages reporting use 1–2 times (48.8% each), followed by cocaine (45.0%), and methamphetamine (42.9%). Among students reporting current alcohol use or current binge drinking, more than half of students (54.8% and 61.2%, respectively) reported those behaviors on 1–2 days. Among students who had ever injected drugs (1.2%), 47.8% reported injecting drugs 1 time, and 52.2% reported injecting drugs ≥2 times.

**TABLE 3:** Frequency of lifetime and current use among high school students reporting use of specific substances — Youth Risk Behavior Survey, United States, 2019

Behavior	Frequency			
	1–2 times	3–9 times	10–39 times	≥40 times
	% (95% CI)	% (95% CI)	% (95% CI)	% (95% CI)
Current use*				
Marijuana (n = 2,946)	36.7 (33.7–39.8)	21.8 (19.7–24.1)	23.5 (21.4–25.8)	18.0 (15.1–21.3)
Prescription opioid misuse (n = 661)	53.2 (47.9–58.5)	23.3 (18.9–28.3)	13.7 (11.0–16.9)	9.8 (6.3–14.8)
**Lifetime use**				
Marijuana (n = 4,219)	24.6 (22.4–27.1)	20.9 (19.3–22.6)	20.8 (19.2–22.5)	33.6 (30.5–36.9)
Prescription opioid misuse (n = 2,000)	48.8 (45.7–51.9)	24.7 (22.4–27.2)	15.9 (14.0–18.1)	10.6 (8.8–12.7)
Synthetic marijuana (n = 955)	48.8 (44.9–52.6)	20.8 (17.8–24.3)	18.5 (14.9–22.8)	11.9 (9.2–15.3)
Cocaine (n = 557)	45.0 (38.7–51.5)	20.3 (15.7–25.9)	18.5 (14.4–23.6)	16.1 (11.8–21.7)
Methamphetamine (n = 351)	42.9 (34.7–51.5)	15.5 (10.8–21.7)	13.7 (9.0–20.3)	27.9 (19.1–39.0)
Heroin (n = 316)	31.7 (24.4–40.1)	18.6 (12.8–26.2)	16.9 (11.8–23.6)	32.9 (21.7–46.3)

Behavior	Frequency			
	1–2 days	3–9 days	10–19 days	≥20 days
	% (95% CI)	% (95% CI)	% (95% CI)	% (95% CI)
**Current use**				
Alcohol use (n = 3,669)	54.8 (52.6–57.0)	36.6 (34.4–38.8)	5.1 (4.1–6.4)	3.5 (2.6–4.7)
Binge drinking (n = 1,657)	61.2 (56.5–65.7)	31.1 (27.7–34.7)	4.1 (2.7–6.2)	3.6 (2.4–5.3)
**Behavior**	Frequency			

1 time	≥2 times
% (95% CI)	% (95% CI)
**Lifetime use**		
Injection drug use (n = 200)	47.8 (35.4–60.4)	52.2 (39.6–64.6)

**Abbreviation:** CI = confidence interval.

*Previous 30 days before the survey.

Students reporting current prescription opioid misuse commonly indicated use of other substances (Figure). Overall, 43.5% of students reporting current prescription opioid misuse also reported current marijuana use, 59.4% reported current alcohol use, and 30.3% reported current binge drinking. Lifetime use of other substances among students reporting current prescription opioid misuse was 62.9% for marijuana, 30.3% for synthetic marijuana, 20.5% for cocaine, 15.0% for methamphetamine, and 14.0% for heroin. Approximately 12.4% of students reporting current prescription opioid misuse also reported lifetime injection drug use.

**FIGURE:**
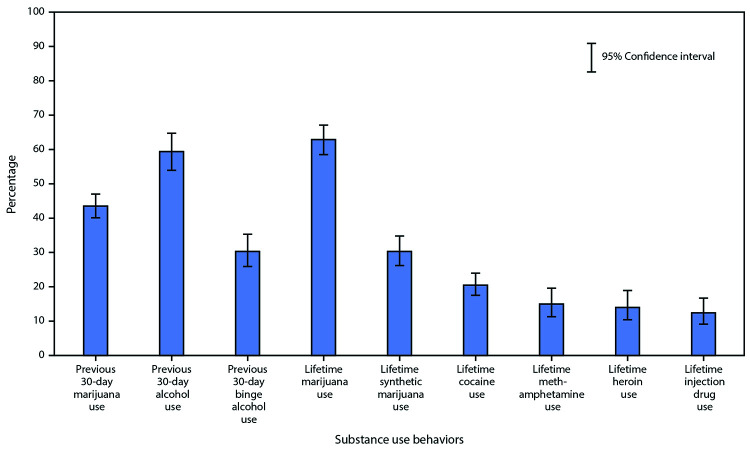
Percentage of co-occurring substance use behaviors among high school students who reported previous 30-day prescription opioid misuse* — Youth Risk Behavior Survey, United States, 2019 *Unweighted N = 661.

In adjusted analyses, current prescription opioid misuse varied by sex, race/ethnicity, and sexual identity (Table 4). Specifically, males were significantly less likely to report engaging in current prescription opioid misuse (aPR: 0.69; 95% CI: 0.57–0.84) compared with females (referent group); black and Hispanic students were significantly more likely to have engaged in prescription opioid misuse (black students, aPR: 1.49; 95% CI: 1.05–2.10; Hispanic students, aPR: 1.52; 95% CI: 1.12–2.05) compared with white students (referent group); and students identifying as lesbian, gay, or bisexual were more likely to report current prescription opioid misuse (aPR: 1.35; 95% CI: 1.02–1.79) compared with students identifying as heterosexual (referent group). All substance use behaviors included in the model, except for marijuana use, were significantly associated with current prescription opioid misuse, ranging from aPR = 2.13 (95% CI: 1.59–2.86) for lifetime synthetic marijuana use and aPR = 2.13 (95% CI: 1.58–2.86) for previous 30-day binge drinking, to aPR = 5.08 (95% CI: 2.72–9.49) for lifetime injection drug use.

**TABLE 4:** Multivariable logistic regression model examining individual-level characteristics associated with previous 30-day prescription opioid misuse among high school students —Youth Risk Behavior Survey, United States, 2019

Characteristic		Adjusted* prevalence ratios (95% CI)
**Demographics**		
Sex		
Female		Referent
Male		0.69 (0.57–0.84)
Race/Ethnicity		
White, non-Hispanic		Referent
Black, non-Hispanic		1.49 (1.05–2.10)
Hispanic		1.52 (1.12–2.05)
Grade		
9 or 10		Referent
11 or 12		0.85 (0.66–1.10)
**Sexual identity**		
Heterosexual		Referent
Lesbian, gay or bisexual		1.35 (1.02–1.79)
Not sure		1.37 (0.86–2.17)
**Substance use and use behaviors**		
Alcohol use		
No previous 30-day use	Referent	
Previous 30-day nonbinge drinking	2.28 (1.63–3.19)	
Previous 30-day binge drinking	2.13 (1.58–2.86)	
Marijuana use		
No lifetime use	Referent	
Lifetime use, but no previous 30-day use	1.21 (0.89–1.65)	
Previous 30-day use	1.31 (0.95–1.80)	
Lifetime synthetic marijuana use		
No		Referent
Yes		2.13 (1.59–2.86)
Lifetime use of cocaine, heroin, or methamphetamine		
No		Referent
Yes		2.49 (1.89–3.27)
Lifetime injection drug use		
No		Referent
Yes		5.08 (2.72–9.49)

**Abbreviation:** CI = confidence interval.

* Adjusted prevalence ratios were calculated from a single logistic regression model that included all covariates listed in this table.

## Discussion

This report provides key insights into substance use behaviors of U.S. high school students during 2009–2019. Encouraging findings include decreasing prevalence of current alcohol use and decreases in the prevalence of lifetime use of marijuana, cocaine, methamphetamine, heroin, synthetic marijuana, and injection drug use. However, the findings in this report underscore that substance use among high school students remains common, with approximately one in three students reporting current alcohol use, one in five reporting current marijuana use, and one in seven reporting current binge drinking. Because of the ongoing U.S. opioid crisis, of particular concern are the high rates of lifetime (one in seven students) and current prescription opioid misuse (one in 14 students) and high rates of co-occurring substance use among students currently misusing prescription opioids.

Notable demographic differences and patterns in substance use among high school students are identified in this report. Specifically, males had substantially higher rates of cocaine, methamphetamine, heroin, and injection drug use compared with females, and females had substantially higher rates of current alcohol use and current binge drinking. In addition, females had higher rates of current prescription opioid misuse compared with males, and this pattern persisted in multivariable models where males had lower adjusted prevalence ratios for current prescription opioid misuse compared with females. Differences also occurred in substance use patterns across racial/ethnic groups. For example, black and Hispanic students reported higher rates of current prescription opioid misuse compared with white students; in contrast, white students reported the highest rates of current alcohol use and binge drinking, followed by Hispanic and black students. These substance use patterns by racial/ethnic groups are similar to those identified in other U.S. youth substance use surveys (https://www.samhsa.gov/data/report/2018-nsduh-detailed-tables). This heterogeneity in substance use patterns among demographic groups can be used to guide development of tailored and targeted prevention messages and interventions.

Particularly noteworthy were the universally elevated rates of substance use among self-identified sexual minority youths compared with heterosexual youths, which is consistent with previous research (*12*). In addition to findings regarding broader substance use patterns, this report provides actionable information on prescription opioid misuse among high school students that can be applied to ongoing efforts for preventing opioid misuse, use disorders, and overdoses. Specifically, the high rates of co-occurring substance use, especially alcohol and marijuana use, among students currently misusing prescription opioids highlights the importance of prevention efforts that focus on general substance use risk and protective factors. Notably, these associations are not limited to high school students because binge drinking and marijuana use are associated with increased prescription opioid misuse among both adults and adolescents (*13*). Finally, sexual minority youths also had significantly higher prevalence of current prescription opioid misuse even after controlling for other demographic and substance use characteristics, which is consistent with their overall pattern of higher rates of substance use in this study. It also further emphasizes the importance of identifying tailored prevention strategies to address disparities among this vulnerable population.

Scientific evidence for the effective prevention of substance use indicates the importance of interventions that target risk and protective factors at the individual, family, and community levels to maximize their public health impact (*1*–*3*). Risk factors include adverse childhood experiences at the individual level, limited parental monitoring and involvement and active substance use in the home at the family level, and easy availability and accessibility to alcohol and other substances and community norms favorable toward use of alcohol and other substances at the community level (*1*,*2*,*5*). In addition, studies have demonstrated that youth alcohol use is associated with adult alcohol use, and that both community-level and individual-level alcohol use are affected by population-level alcohol policies (e.g., those that reduce the availability and accessibility of alcohol and increase its price) (*14*).

The ability to reach young persons during early elementary ages, before they begin using substances, and throughout adolescence makes the school environment well-suited for prevention programming. School-based substance use prevention programs that focus on broad-based skill building (e.g., psychosocial development, life-skills development, and social-emotional learning and connectedness) have greater promise than substance-specific programs (*15*,*16*). In addition, multifaceted programs that incorporate aspects of individual, school, and family interventions (e.g., the Promoting School-community-university Partnerships to Enhanced Resilience [PROSPER] program and Communities That Care [CTC]) have demonstrated effectiveness at reducing or preventing youth substance use (*17*,*18*).

Broader prevention policies for changing the environment in which youths live (e.g., those that reduce the availability of substances) can also be used as part of a comprehensive approach for reducing youth substance use. The U.S. Community Preventive Services Task Force recommends certain population-level strategies (e.g., increasing alcohol taxes and regulating the number and concentration of places that sell alcohol as interventions for reducing excessive alcohol use, including alcohol use among youths) (https://www.thecommunityguide.org/resources/what-works-preventing-excessive-alcohol-consumption). Enhanced enforcement of existing substance use policies (e.g., prescription drug monitoring programs that are used universally with near–real-time data and laws prohibiting sales of alcohol to persons aged <21 years) also can help reduce substance use among youths (*19*,*20*). In addition, strategies for expanding access to evidence-based pain treatment and improving prescribing of prescription opioids through safer prescribing practices can help reduce opioid misuse and overdoses. Improving opioid prescribing can have dual benefits by reducing the environmental availability of prescription opioids for diversion and misuse and reducing the risk for misuse associated with the prescription of opioids to youths (*2*).

## Limitations

General limitations for the YRBS are available in the overview report of this supplement (*11*). The findings in this report are subject to at least three additional limitations. First, the questions assessing lifetime and current prescription opioid misuse refer to prescription pain medicine; however, the questions provide examples of opioid-containing prescription medications only. Therefore, if students considered nonopioid prescription pain medications when answering, an overestimation of prescription opioid misuse prevalence might have occurred. Second, many of the substance use questions included common street names for drugs; however, newly introduced street names or street names specific to certain geographic areas were not included, which might have resulted in underreporting of substance use behaviors. Finally, there was variation in the amount of missing data for some substance use variables (e.g., the largest amount missing was for current prescription opioid misuse [5,000 missing observations]). Missing data might result from a variety of factors, such as students choosing not to answer questions or inconsistent responses to similar questions that are set to missing during the data cleaning process (*11*). In addition, schools selected to participate in the national YRBS and in a state or local YRBS only complete the local version of the survey; as a result, questions included on the national survey but not the local survey are set to missing.

## Conclusion

The findings in this report indicate that youth substance use has declined in recent years; however, substance use, including misuse of prescription opioids, remains common among U.S. high school students. Opportunities exist for bringing to scale evidence-based policies, programs, and practices that aim to reduce risk factors and strengthen protective factors among youths in conjunction with initiatives already underway for combating the U.S. opioid overdose epidemic. Disproportionately affected populations (e.g., sexual minority youths) might benefit from tailored substance use interventions combined with more widespread implementation of broader population-level policy strategies.
